# Integrated geospatial datasets to inform marine spatial planning and impact assessment in waters surrounding the United Kingdom

**DOI:** 10.1038/s41597-025-05950-5

**Published:** 2025-11-20

**Authors:** Hugo Putuhena, Thomas J. Williams, Fraser Sturt, David White, Martin Solan, Jasmin A. Godbold, Susan Gourvenec

**Affiliations:** 1 Civil, Maritime, and Environmental Engineering, Boldrewood Innovation Campus, University of Southampton, Burgess Road, Southampton SO16 7QF UK; 2 Ocean and Earth Science, National Oceanography Centre Southampton, University of Southampton, Waterfront Campus, European Way, Southampton SO14 3ZH UK; 3 Archaeology, Avenue Campus, University of Southampton, Highfield Road, Southampton SO17 1BF UK

**Keywords:** Environmental impact, Geography, Biodiversity

## Abstract

The rapid expansion of human activity in coastal and shelf seas provides impetus to investigate increased risks to ocean health and social-ecological resilience, but progress in understanding the role and relative importance of associated pressures is frustrated by a lack of a routinely available set of processed geospatial information. Here, we pool 337 standardised geospatial layers derived from 35 sources, including anthropogenic activities, ecological and geoscience assets and features, and met-ocean conditions for the exclusive economic zone surrounding the United Kingdom. Our compilation has undergone pre-processing: spatial interpolation, density estimation, data resampling and extraction, and harmonisation to populate ~10 km^2^ grids. We provide source version history, an open-access interactive portal, and the details of the spatial processes we used to create each layer, including data quality and uncertainties for layers generated by interpolation/density estimation. Our motivation is to provide reference information and spatio-temporal context, encourage the exploration and inclusion of any inter-dependencies between layers when determining system response, improve mechanistic understanding of observed patterns, and enable better parameterisation of models for those tasked with assessing the compound and cumulative effects of anthropogenic activity.

## Background & Summary

The blue acceleration^1^ – the unchecked advancement of diverse and competing interests for ocean-based food, resources, and space – is placing increasing pressure on the world’s oceans and seas. Whilst plans for fishing, aquaculture, renewable energies, designated protected areas, and other human benefits are being realised at pace^1^, so too is our knowledge of, and commitment to, protection of the marine environment and cultural assets; from the location and status of tangible and intangible heritage sites^2^ through to improved understanding of marine biodiversity and ecosystem functioning^3,4^. The principal way of ensuring that development and environmental protection needs are both satisfied as best as possible is through marine spatial planning and environmental impact assessment, yet the wider context of an activity or pressure in time and space is seldom considered.

Recent years have seen a rapid evolution in attitudes relating to marine spatial planning, with integrated approaches gaining traction^5^. The data required to represent and holistically understand the complexity of marine and maritime space in support of this activity is frequently available, but often in divergent formats, at varying scales, resolutions and/or lodged in technically demanding repositories that are only familiar to siloed specialist research areas^6^. Meanwhile, major data gaps remain in our understanding of anthropogenic activity – ecosystem interactions^7^, highlighting the urgent need to consolidate and increase the accessibility of available data to widen the reach of current knowledge and ensure future collection efforts are appropriately targeted. Consequently, there are few examples of system-scale overviews of the status and condition of the marine environment (e.g., Scottish waters^8^) on which to base management and policy decisions.

Here, motivated by the need to address the challenges caused by the constrained availability and fragmented nature of data and knowledge, we use a case study of UK waters to demonstrate how a wide range of candidate correlates can be combined and integrated to provide a clear overview of the use, condition and status of the shelf seas at a national scale. We provide a single data repository, offering integrated geospatial datasets encompassing 337 spatial layers covering the exclusive economic zone surrounding the United Kingdom (hereafter, UK-EEZ). The layers were selected by an interdisciplinary team and span various themes, including anthropogenic features (such as offshore infrastructures, vessel operations, fisheries, and heritage assets), ecological parameters (including seafloor biodiversity and ecosystem process and function), geoscience features and met-ocean conditions to capture salient aspects of the seabed and ocean environments. The resulting spatial layers derived from the 35 source layers are outlined in Table 1, each of which has been processed and integrated to populate 10 km^2^ grids across the UK-EEZ, offering considerable savings in time for the user by eliminating the need to gather and process existing datasets from multiple sources. We document the spatial processes employed to generate each layer, including uncertainties produced during spatial processing, thereby providing full traceability and allowing for further assessment or evaluation of data quality.

**Table 1:** List of collected datasets.

	List of original data classified into the category	Data format type	Time series [year window]	Data code	Ref.
Anthropogenic theme	AIS tracked lines for each vessel per year	(ii)	[2011–2020]	D-A1	^20^
	Pulse block days of anthropogenic noises	(ii)	[2015–2021]*	D-A2	^21^
	Global satellite observation of operational turbines	(ii)	[2003–2020]	D-A3	^22^
	Shipwrecks, heritage assets, and obstructions	(ii)	No [-]	D-A4	^23^
	Subsea power and telecommunications	(ii)	No [-]	D-A5	^24,25^
	Oil and gas infrastructures	(ii)	No [-]	D-A6	^26^
	Active offshore wind sites and cables	(iii)	[2003–2022]	D-A7	^27–29^
	Palaeolandscape sites	(iii)	[-]	D-A8	^30^
	Restricted blocks associated with the Ministry of Defence (MoD) activities	(iii)	[-]	D-A9	^31^
	Fishing effort for total fishing	(iv)	[2007–2020]*	D-A10	^32^
	Satellite observation: vessel and infrastructure data	(ii)	[2017–2022]*	D-A11	^33^
	Fishing effort for trawlers or non-trawlers gear vessels	(ii)	[2012–2020]	D-A12	^34^
Ecological theme	Benthic sampling data	(i)	[2000–2020]**	D-E1	^35,36^
	Marine protected areas	(iii)	[1971–2020]	D-E2	^37^
	Bioturbation intensity	(iii)	No [-]	D-E3	^38^
	Benthic mixed depth layer	(iii)	No [-]	D-E4	^38^
Geoscience theme	Offshore geotechnical data	(i)	No [-]	D-G1	^39^
	Offshore seabed sediment data	(i)	No [-]	D-G2	^39^
	Sub-glacial bedforms features	(ii)	No [-]	D-G3	^40^
	Seabed sediment characteristics	(iii)	No [-]	D-G4	^41–45^
	Seabed sediment type	(iv)	No [-]	D-G5	^46^
	Various parameters on the seabed [Bio-oracle v.2]	(iv)	No [-]	D-G6	^47^
	Predictor variables and ground truth samples	(iv)	No [-]	D-G7	^48^
	Quantitative sediment composition (geoscience data)	(iv)	No [-]	D-G8	^48^
	Predicted sediment and physical parameters	(iv)	No [-]	D-G9	^49^
	Density, carbon, and porosity of the seabed	(iv)	No [-]	D-G10	^50^
	Various parameters on the seabed [Bio-oracle v.3]	(iv)	No [-]	D-G11	^51^
Met-ocean theme	Current and wave kinetic energy on the seabed	(iii)	No [-]	D-MO1	^52^
	Wind speed data	(iv)	No [-]	D-MO2	^53^
	Wave and tidal data	(iv)	No [-]	D-MO3	^54^
	Water depth data	(iv)	No [-]	D-MO4	^55^
	Quantitative sediment composition (met-ocean data)	(iv)	No [-]	D-MO5	^48^
	Adverse weather scenarios	(iv)	No [-]	D-MO6	^56^
	Sea surface waves [RisesAM-NEA-clim]	(iv)	No [-]	D-MO7	^57^
	Kinetic energy from waves and currents	(iv)	No [-]	D-MO8	^58,59^

*) The year window given is the maximum range given from the data in the data source; each data may have a different year window.

**) The data source contains the older sampling data from the year window stated. But for this paper, the data sampling used is constrained to the year window given.

The dataset allows the study of interactions between specific layers, or layers within themes, as relevant to anthropogenic activity and/or the implementation of alternative management strategies, policy regimes or other scenarios of interest.

The integrated layers provided here form a valuable resource for future marine spatial planning and impact assessment^9^, and for those tasked with balancing human intervention and pressures to meet societal demands with the protection of ocean health. Primary uses of these data, for example, could include, but are not limited to: (1) assessment of spatial patterns and temporal trends in anthropogenic pressures, and their interactions, with respect to their potential implications for seabed condition, (2) exploration of ecological changes across different regions and timeframes using environmental and benthic indicators, (3) investigation of relationships between human use of marine space and the delivery or degradation of benthic biodiversity, habitat, and ecosystem functioning, (4) examination of anticipated or potential future scenarios to understand how projected human activities may influence seabed ecology, or they may be used to, (5) support the identification of knowledge gaps and inform prioritisation for future ecological monitoring and data collection efforts.

These data can be visualised and explored via an online Geographic Information System (GIS) dashboard (https://storymaps.arcgis.com/collections/0f2956eee9704625b74d5cc6157a879d).

This work presents three novel aspects to the integration of geospatial data in the context of human activities and impact assessments—and forms an approach which, to our knowledge, has not previously been implemented in the UK-EEZ or globally:

1. **Provision of a comprehensive, multidisciplinary geospatial dataset** of 337 data layers covering anthropogenic, ecological, geoscience and met-ocean themes, constructed from a total of 35 harmonised and integrated data sources (Table 1) and used to populate regional-scale grid cells (~10 km^2^).
2. **Inclusion of time series data for key layers** (e.g., human activities and seafloor ecological parameters), with annual time steps spanning from 2000 to 2020, noting that not all layers include time series data, and discrepancies exist in temporal coverage across datasets (further discussed in the Usage Notes).
3. **Generation of continuous spatial layers from sampling datasets with spatial gaps** using empirical Bayesian kriging (EBK)^10^, a method which is considered more robust than other interpolation methods due to its ability to model local spatial correlation and quantify uncertainty^11–13^.

## Methods

**Fig. 1:**
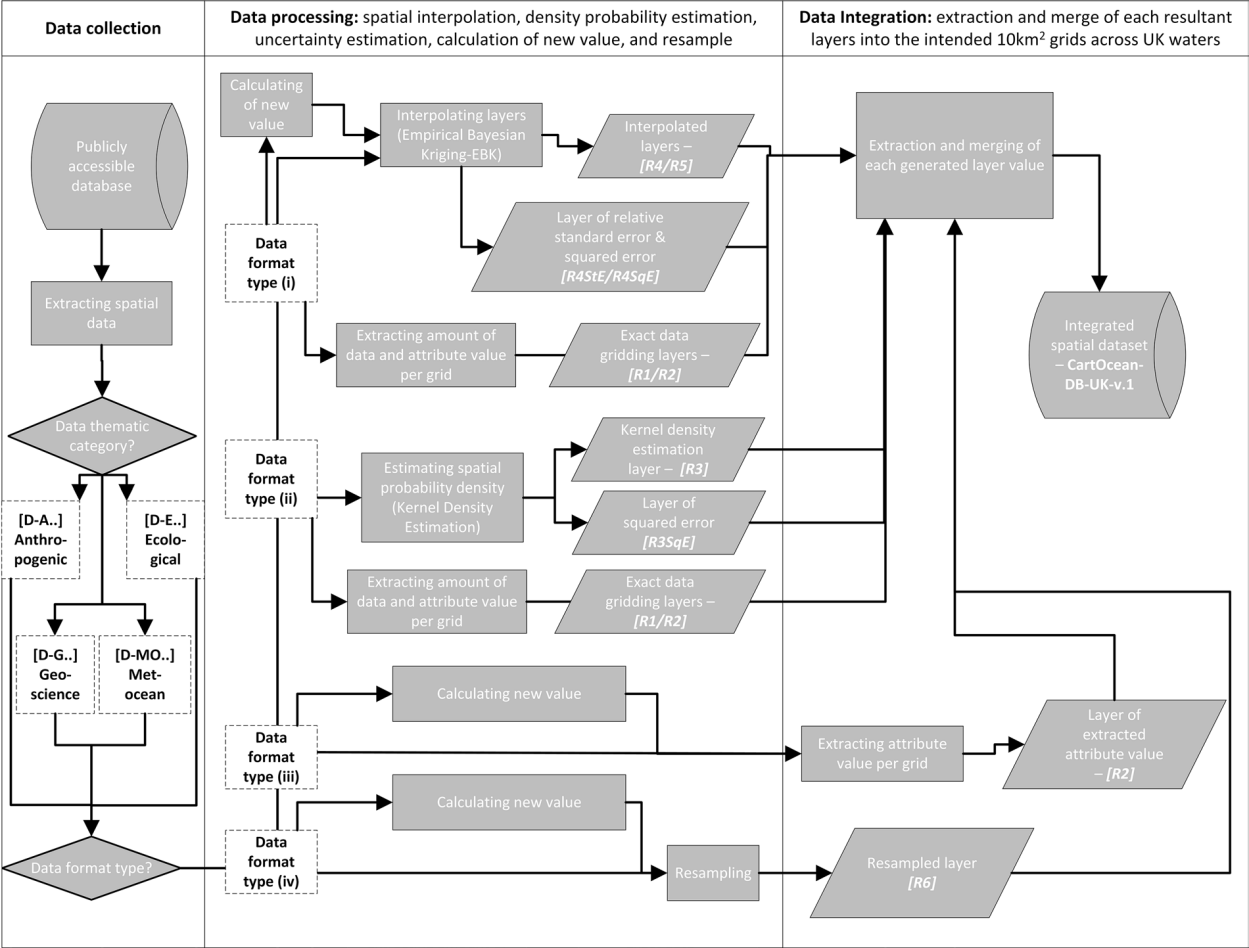
Summary of methodological sequence to generate the geospatial dataset, from data collection, processing, to the integration.

A high-level workflow for the data collection, processing, and integration that we used to generate the dataset is illustrated in Fig. 1.

### Data collection

The four thematic domains of data (Table 1) that were collected and/or generated for this dataset are: *Anthropogenic* – covering historical human activities across the region over multiple years (12 data sources, 100 layers); *Ecological* – consisting seafloor ecological parameters and marine protected areas (4 data sources, 123 layers); *Geoscience* – encompassing physical/biological/chemical/geological seabed characteristics that may affect the seafloor ecology (11 data sources, 94 layers); or *Met-ocean* – covering physical parameters of the atmosphere and water column (8 data sources, 20 layers). All the data are available from public domain sources and are under licenses that allow data derivations for data sharing purposes—see Supplementary Information 2. All 337 generated layers are summarised in Supplementary Information 2.

Each data source is identified with a data format type, which includes (i) point/polyline/polygon vectors, each representing a single, discrete, and specific geographic location with single or multiple unique sets of continuous numeric attribute values, data format type (ii) data in multi-points/polylines/polygons vectors, each representing a single, discrete, and specific geographic location of a certain single entity, data format type (iii) data in polygon vectors that each represent a single, discrete, and specific geographic zonation with some unique sets of classified attribute values, or data format type (iv), data in raster or gridded polygon vectors populated with a single or multiple continuous numeric attribute values—to understand whether data gridding, kernel density estimation, spatial interpolation or resampling was needed (Supplementary Information 1).

Thirteen different source data, and 100 generated layers from them, out of a total of 337 generated layers, are attributed to a time dimension. These layers are in the anthropogenic (e.g., vessel operations, noise, fishing, and offshore infrastructure) or ecological (e.g., benthic biodiversity and function parameters) theme. Layers generated without a time dimension, either portray a spatial condition from a single observation in time or a statistical condition across a specific time range. These layers are either in the anthropogenic (e.g., shipwrecks, seabed obstruction, and oil and gas infrastructure), ecological (e.g., bioturbation intensity, benthic mixed depth), geoscience (e.g., geotechnical and seabed data), or met-ocean (e.g., water depth, wave, wind, and currents) theme.

### Data processing

The primary objective of data processing is to convert the source data to populate the underlying square grids (@10 km^2^ resolution) across the UK-EEZ, calculate standard error/uncertainties, and calculate new values from the source data given. Each data format type requires the application of different data processing methods to produce the relevant layers.

Eleven different types of resultant layers *[R]* can be generated across the different data types, as illustrated in Fig. 1: extracted amount of point/polyline *[R1]*, extracted attribute value *[R2]*, kernel density estimation *[R3]*, and squared error *[R3SqE]*, interpolation without limit zone *[R4]* with the standard error *[R4StE]*, and squared error *[R4SqE]*, interpolation with limit zone *[R5]* with the standard error *[R5StE]*, and squared error *[R5qE]*, and the resampled value *[R6]*.

**Fig. 2:**
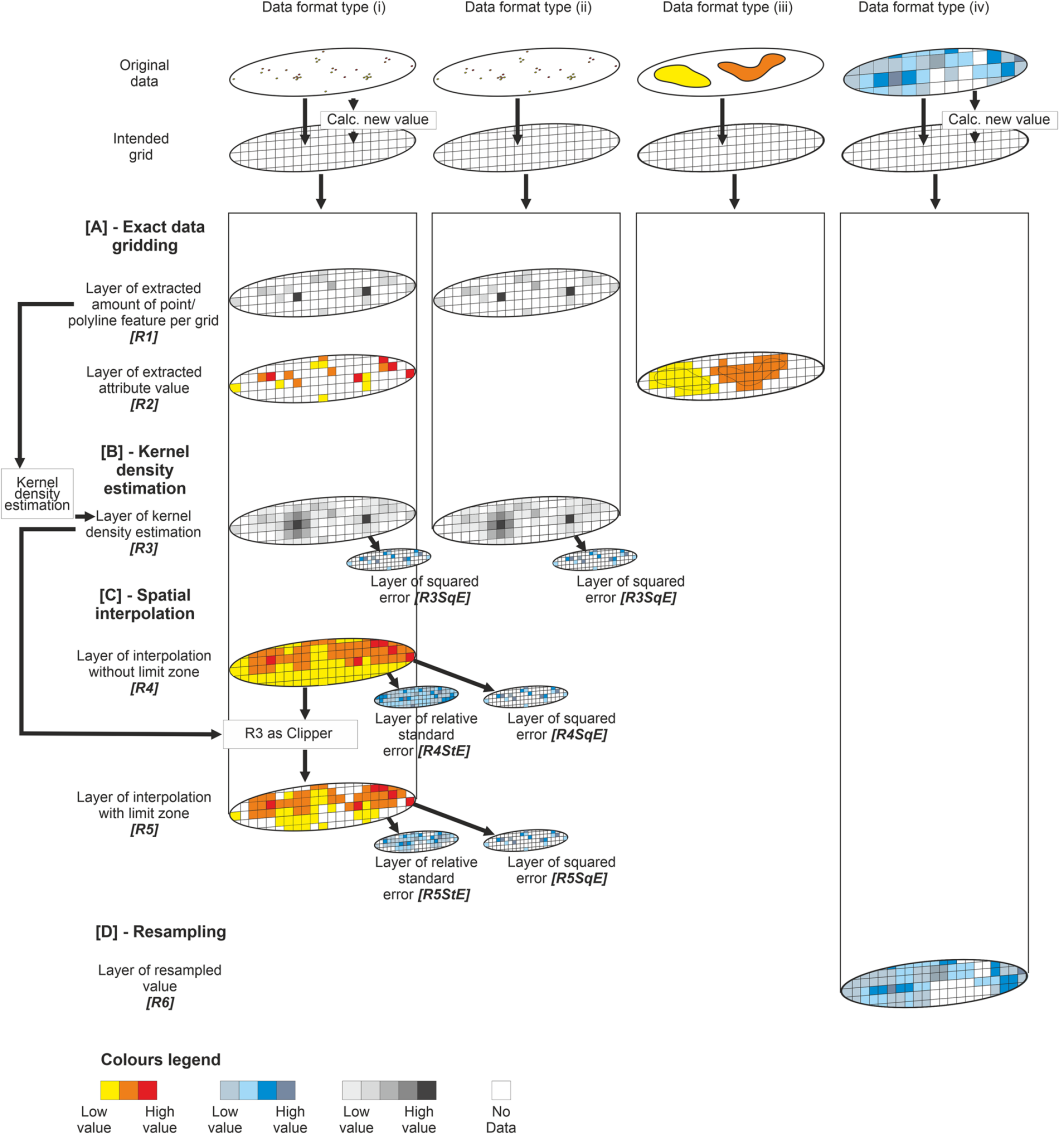
Summary of data processing for the different source data format types encountered across the input data, used to generate layers in the integrated dataset. As described in the figure, depending on the data format types (from Type i to iv, see manuscript for further descriptions), each input data underwent a range of methods, from [**A**] exact data gridding, [**B**] kernel density estimation, [**C**] spatial interpolation, to [**D**] resampling to fill in the intended 10 km^2^ grids.

The key processing steps to generate the resultant layers, as summarised in Fig. 2, are:

A. *Data Gridding*. Data gridding was used to populate attribute values or amounts from source data format type (i) and (ii) into each cell grid. This involved assigning statistical values (e.g., the mean of benthic ecological parameters or the total number of samples) to each ~10 km^2^ grid cell, based on available data samples. Grids were created using the ED50 UTM 30 N projection, which offers a spatial accuracy of 1–5 m (https://epsg.io/23030), sufficient for regional-scale mapping. This method also allows exact density calculation for each grid cell (e.g., to calculate the density of AIS track vessels or sample counts).
B. *Kernel density estimation*. Kernel density estimation^14^ was used to create an alternative visualisation of density layers from point, polyline, or polygon source format type for visual comparison to the density calculation generated from the exact gridding value. This method uses kernel functions to smooth out data density, generating a continuous curve that reflects the probability density.
C. *Spatial interpolation*. Empirical Bayesian kriging (EBK) interpolation was used to generate a continuous spatial data layer from point-sample datasets that contained spatial gaps. We generated two resultant layers, spatial interpolation layers with *[R5]* and without *[R4]* a limit zone. The interpolations of layers with a limit zone were limited to within an area of 2–3 grid lengths of where sampling exists. This was applied to source data format type (i), in particular benthic ecological samples with different attributed values to interpolate (i.e., mean bioturbation potential index (BPc), Shannon diversity index, mean mobility mode (Mi), mean reworking mode (Ri), species evenness, species richness, total abundance, mean body mass, and biomass). The interpolations of layers without a limit zone, extend to a rectangular zone generated from the outermost sampling points. This interpolation was also conducted on the benthic ecological data mentioned above, for comparison purposes with the interpolations without a limit zone, and to seabed and geotechnical data (i.e., compressive strength, shear strength, percentage carbonate in mud, sand, gravel, and total sediment).
  Different data transformations were conducted during the interpolation for each layer, depending on whether the data is normal and whether the data contains negative or zero values. See Supplementary Information 1 for more detail on how the data transformation for each layer was decided and which data transformation were carried out in the interpolation.
D. *Data resampling*. To ensure consistency across all integrated layers, we resampled source data that were originally continuous but differed in resolution from the target grid size (~10 km^2^).

### Data integration

All resultant layers were then extracted to fill in 10 km^2^ gridded cells across the UK-EEZ^15^, and merged to create two main datasets: the layers with a time dimension and those without a time dimension.

Details of each processing method are provided in Supplementary Information 1, while the specific method used for each dataset layer is compiled in Supplementary Information 2.

All methods in this sequence were conducted using ArcGIS Pro (version 3.3).

**Fig. 3:**
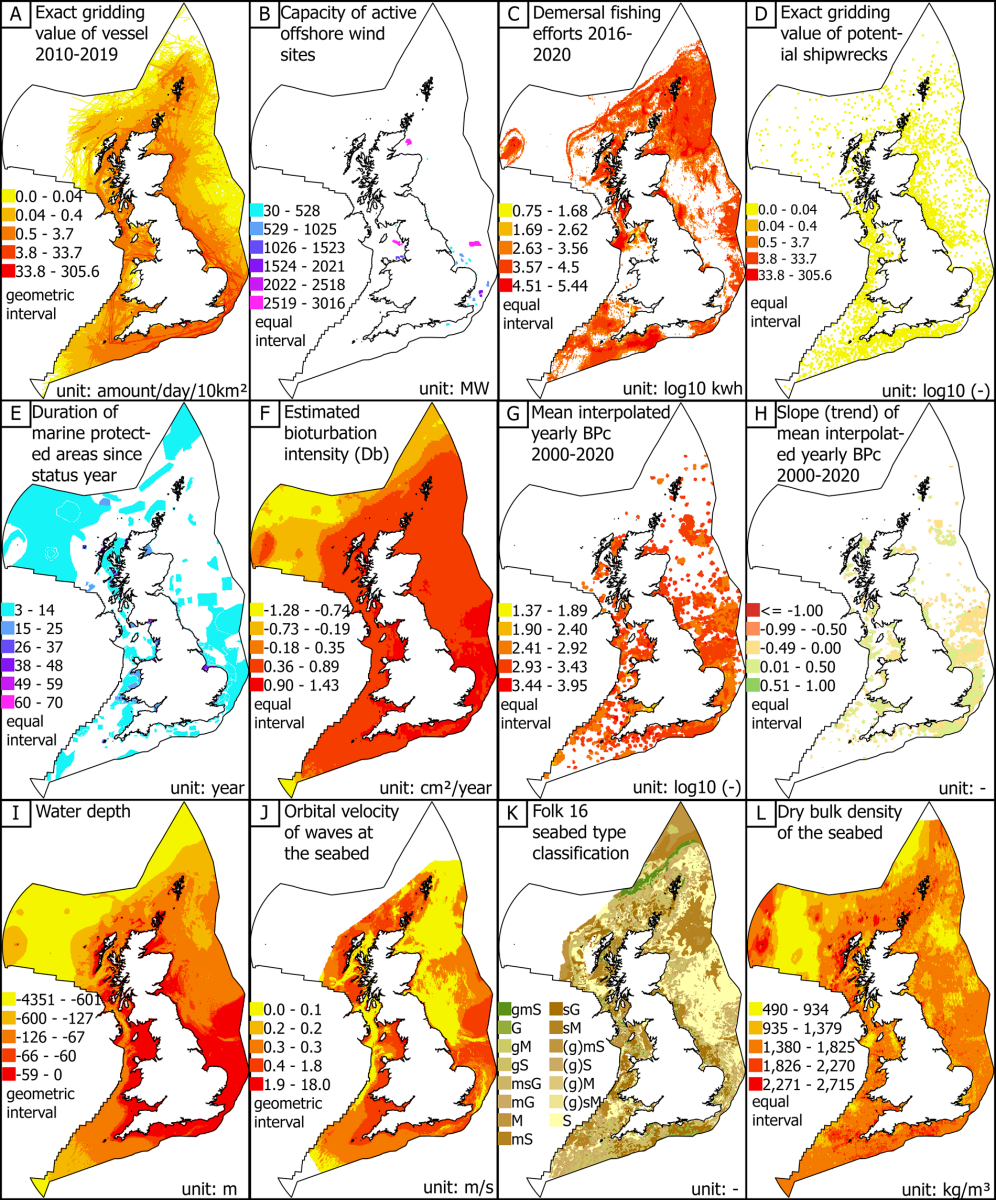
A selection of twelve generated layers across different themes: [**A**–**D**] anthropogenic, [**E**–**H**] ecological, [**I**,**J**] met-ocean, and [**K**,**L**] geoscience. Each map includes notations specifying the represented layer, units, and colour bar interval type. Data sources and further details are provided in Supplementary Information 2. (Abbreviations: km – kilometers, MW – megawatt, kwh – kilowatt-hour, BPc—Bioturbation index, cm—centimeters, m—meter, s—seconds, kg—kilograms).

For illustration, we present a selection of generated spatial layers across the four different themes in Fig. 3. Graphical representations of all spatial layers are included in Supplementary Information 3.

## Data Record

The dataset is openly available without restriction at the University of Southampton Pure data repository^16^. We separate individual layers into one of two distinct datasets based on whether or not there was a temporal dimension attribution and provide detailed information for each layer in Supplementary Information 2. We also provide these data as an open GIS dashboard accessed via https://storymaps.arcgis.com/collections/0f2956eee9704625b74d5cc6157a879d.

## Technical Validation

From the total 337 layers in this integrated geodataset, there are 33 interpolation layers (15 interpolation layers with no time series, 9 interpolation layers with time series and limit zone, and 9 interpolation layers with time series and no limit zone), and 26 kernel density estimated layers (10 estimation layers of no time series, and 16 estimation layers of time series) that are not just extracted or resampled from observation/model data given in the data source, and as such require technical validation. For validation, we calculated the mean relative standard error (RSE) to understand the uncertainty of the resulting interpolation layers, and root mean square error (RMSE) along with coefficient of determination (R^2^) to measure the deviation of both interpolated and kernel density estimation layers.

**Fig. 4:**
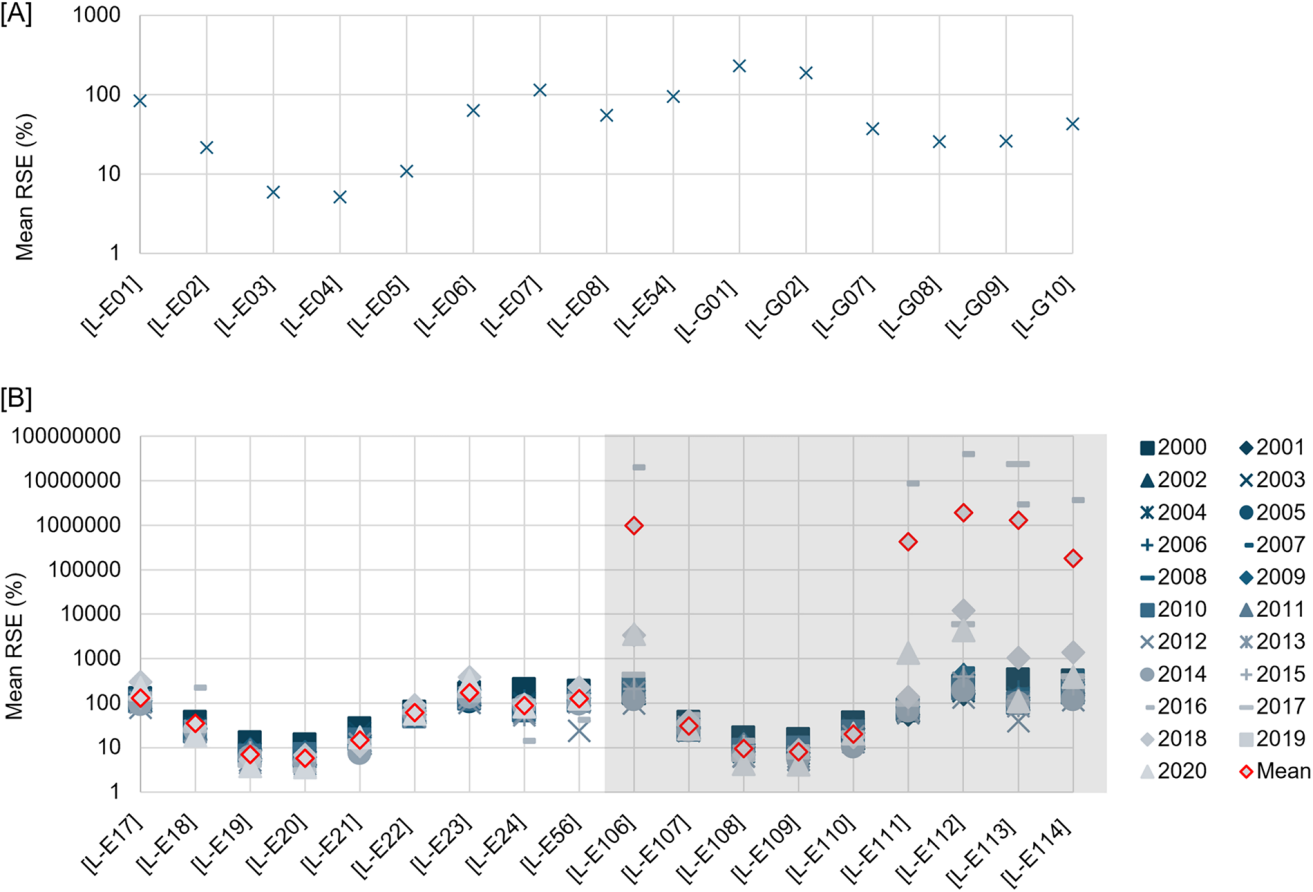
Relative standard error (RSE) for [**A**] interpolated layers with time series and [**B**] interpolated layers without time series. Layers code in **[A]**, [L-E01]: mean bioturbation potential index (BPc), [L-E02]: Shannon diversity index, [L-E03]: mean mobility mode (Mi), [L-E04]: mean reworking mode (Ri), [L-E05]: species evenness, [L-E06]: species richness, [L-E07]: total abundance per meter square, [L-E08]: mean body mass, [L-E54]: biomass per meter square, [L-G01]: compressive strength, [L-G02]: shear strength, [L-G07]: percentage carbonate in sand, [L-G08]: percentage carbonate in mud, [L-G09]: percentage carbonate in gravel, [L-G10]: percentage carbonate in total sediment. Layers code in **[B],** [L-E17, L-E106]: mean bioturbation potential index (BPc), [L-E18, L-E107]: shannon diversity index, [L-E19, L-E108]: mean mobility mode (Mi), [L-E20, L-E109]: mean reworking mode (Ri), [L-E21, L-E110]: species evenness, [L-E22, L-E111]: species richness, [L-E23, L-E112]: total abundance per meter square, [L-E24, L-E113]: mean body mass, [L-E55, L-E114]: biomass per meter square.

**Fig. 5:**
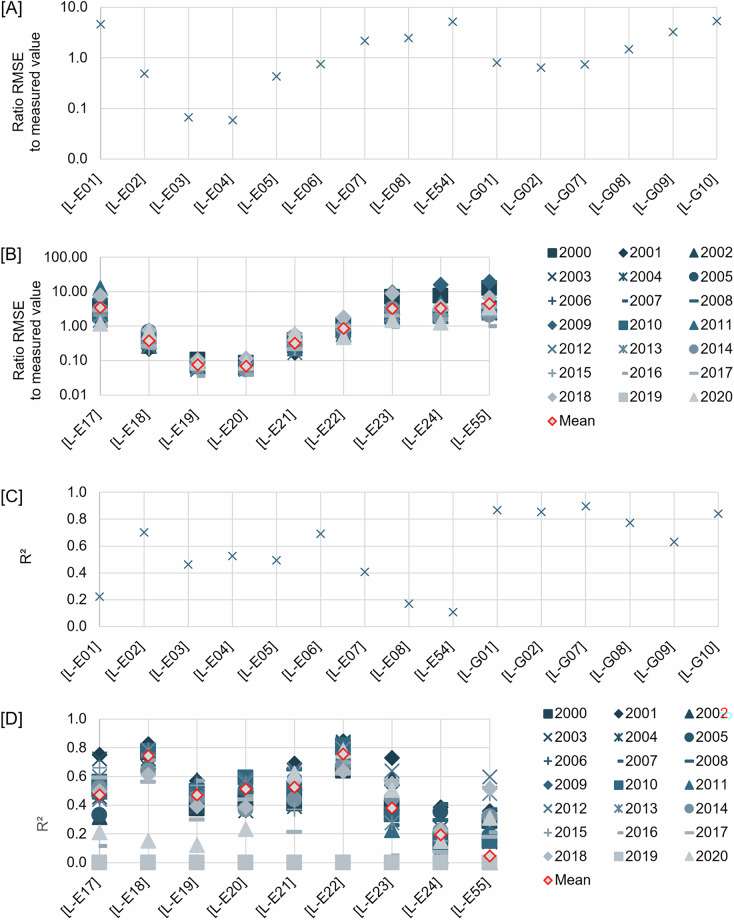
Ratio of the root mean square error (RMSE) of the interpolated layers to the exact gridding mean value for each grid, and the R^2^ between the interpolated layers and the exact gridding mean value for each grid: [**A,C**] interpolated layers with time series and [**B,D**] interpolated layers with no time series, respectively. Layers code in **[A,C]**, [L-E01]: mean bioturbation potential index (BPc), [L-E02]: Shannon diversity index, [L-E03]: mean mobility mode (Mi), [L-E04]: mean reworking mode (Ri), [L-E05]: species evenness, [L-E06]: species richness, [L-E07]: total abundance per meter square, [L-E08]: mean body mass, [L-E54]: biomass per meter square, [L-G01]: compressive strength, [L-G02]: shear strength, [L-G07]: percentage carbonate in sand, [L-G08]: percentage carbonate in mud, [L-G09]: percentage carbonate in gravel, [L-G10]: percentage carbonate in total sediment. Layers code in **[B,D]**, [L-E17]: mean bioturbation potential index (BPc), [L-E18]: Shannon diversity index, [L-E19]: mean mobility mode (Mi), [L-E20] mean reworking mode (Ri), [L-E21]: species evenness, [L-E22]: species richness, [L-E23]: total abundance per meter square, [L-E24]: mean body mass, [L-E55]: biomass per meter square.

**Fig. 6:**
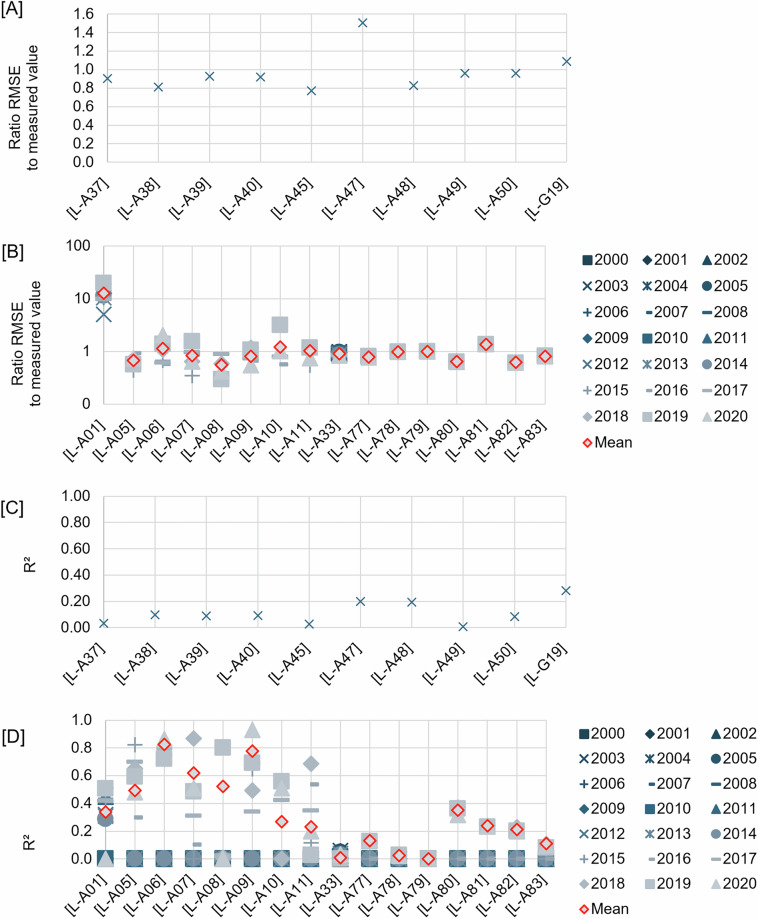
Ratio of the root mean square error (RMSE) of kernel density estimation (KDE) layers to the exact gridding mean value for each grid, and the R^2^ between the KDE layers and the exact gridding mean value for each grid: [**A,C**] KDE layers with time series, and [**B,D**] KDE layers without time series. Layers code in **[A,C]**, [L-A37]: heritage assets - potential shipwrecks, [L-A38]: heritage assets - dangerous shipwrecks, [L-A39]: heritage assets - floating and fixed heritage assets, [L-A40]: heritage assets - obstructions, [L-A45]: subsea power and telecommunications cables, [L-A47]: subsea points, [L-A48]: subsea linear: [L-A49]: pipeline freespans, [L-50]: pipeline, [L-G19]: sub-glacial bedforms features. Layers code in **[B,D]**, [L-A01]: AIS track vessels per day per grid for each year, [L-A05]: noises - seismic survey airguns, [L-A07]: noises - explosion, [L-A09]: noises - sub bottom profiler, [L-A10]: noises - acoustic deterrent device, [L-A11]: noises - piling, [L-A77]: satellite observation - infrastructure wind, [L-A78]: satellite observation - infrastructure oil, [L-A79]: satellite observation - infrastructure unknown, [L-A80]: satellite observation - AIS fishing, [L-A81]: satellite observation - vessel - AIS non fishing, [L-A82]: satellite observation - vessel - dark fishing, [L-A83]: satellite observation - vessel - dark non fishing.

Here we describe the equations of the parameters and graphical representation of the measures. Mean RSE are given in Fig. 4[A] for layers with no time series, and Fig. 4[B] for layers with time series; the ratio of RMSE to the exact gridding value is shown in Fig. 5[A] for layers with no time series, and Fig. 5[B] for layers with time series; and R^2^ is shown in Fig. 5[C] for layers with no time series, and in Fig. 5[D] for layers with time series. The ratio of RMSE to the exact gridding value and R^2^ for the kernel density layers are given in Fig. 6[A] and [C], respectively for layers with no time series, and Fig. 6[B] and [D], respectively for layers with time series.

### Mean Relative Standard Error (RSE)

We include an estimate of uncertainty for all layers generated using empirical Bayesian kriging (EBK) spatial interpolation. Uncertainties are represented by the ratio of the kriging variance given by the semivariograms of the kriging interpolation upon the prediction, and averaged for all interpolated grids, which is termed as the mean relative standard error (RSE) (Eq. 1).

1 $${mean\; RSE}\left( \% \right)=\frac{1}{{\rm{n}}}{\sum }_{i=1}^{n}\frac{{{SE}}_{i}}{{z}_{i}}\ast 100$$

Where: $n$ represents the total grids with interpolation values, and ${SE}$ and $z$ are the standard error or kriging variance from the semivariograms and the interpolation value respectively from the interpolation result for each grid ($i$). Higher mean RSE indicates higher uncertainties.

The uncertainty results for the interpolated layers show that most (n = 12, from 15 layers with no time series, e.g. Shannon diversity index (SDI), species evenness, and percentage carbonate in sand—Fig. 4[A]; n = 10, from 18 layers with time series based on the mean value for all years, e.g. SDI, mean mobility mode (Mi), and mean reworking mode (Ri)—Fig. 4[B]) of the interpolated layers have 0.1 to 2 times that of the standard error range (based on the kriging semivariograms variance) compared to the estimated value (or mean RSE < 100%). But, for some layers (n = 3 for layers with no time series, i.e. total abundance, percentage carbonate in gravel, and compressive strength—Fig. 4[A]; n = 8 for layers with time series, e.g. mean bioturbation potential index (BP_c_), total abundance, and biomass—Fig. 4[B]), the standard error does exceed twice the estimated value (or mean RSE > 100%). Furthermore, the mean RSE also shows that interpolation layers given with constraints for the interpolation zone [L-E17 to L-E54] have a smaller mean RSE than the layers without [L-E106 to L-E114]. The layers with narrower range values (e.g. Species evenness, mean M_i_, BP_c_ in log_10_ layers) also have a lower mean RSE compared to the layers with wider range values (e.g. BP_c_ not in log _10,_ mean body mass, biomass, or total abundance). See Fig. 4 for more details of RSE for each layer.

### Root Mean Square Error (RMSE)

We measured the deviation between the interpolation prediction/kernel density estimation and the mean observed value per grid value using the root mean square error (RMSE) (Eq. 2). For interpolated layers that generated twice with and without zone limit (i.e. the benthic ecological parameters), RMSE was only conducted for one layer since both are basically from the same interpolation results.

2 $${RMSE}=\sqrt{\frac{1}{{\rm{n}}}{\sum }_{i=1}^{n}{{(z}_{i}-{{ze}}_{i})}^{2}}$$

Where: $n$ is the total grids with interpolation, $z$ and ${ze}$ are the interpolated and the exact gridding value extracted from the original data for each grid ($i$) respectively. Higher RMSE indicate higher deviation.

Some of the interpolated layers (n = 8, from 15 layers with no time series, e.g. SDI, Mi, Ri, and seabed compressive strength—Fig. 5[A]; n = 5, from 9 layers with time series based on the mean value for all years, e.g. Mi, Ri, and species evenness—Fig. 5[B]) have the ratio of RMSE to the measured value (or the exact mean extracted per grid) of < 1. While for the rest (n = 7, from 15 layers with no time series, e.g. BPc, total abundance, and mean body mass—Fig. 5[A]; n = 4, from 9 layers with time series based on the mean value for all layers, e.g. BPc, total abundance, and mean body mass—Fig. 5[B]), the RMSE can increase to 10 times the measured value.

While for the kernel density estimation layers, almost all (n = 8, from 10 layers of no time series, e.g. potential shipwrecks, dangerous shipwrecks and subsea power and telecommunications cables—Fig. 6[A]; n = 11, from 16 layers of time series based on the mean value for all layers, e.g. noises echosounder and satellite observation of offshore infrastructures—Fig. 6[B]) have RMSE ratio to the exact mean extracted per grid of < 1. With a few (n = 2, from 10 layers of no time series, i.e. subsea points infrastructures and sub-glacial bedforms—Fig. 6[A]; n = 5, from 16 layers of time series, i.e. AIS track vessels, noises acoustic deterrent device, and satellite observation of offshore infrastructures/vessels) having an average of the RMSE ratio per year data of >1. Nevertheless, it is important to note that: (a) the RMSE is a function of the bandwidth used in the kernel density estimation method^17^, for which we used Silverman’s Rule-of-thumb bandwidth estimation^17^—to note a smaller bandwidth would result in smaller RMSE, and (b) despite low RMSE given (i.e. those layers with ratio RMSE <1), the estimations given outside of the area of where observations exist are less reliable^18^. We provide these estimations to complement, rather than replace, the exact density gridding, providing a smoother version of spatial visualisation.

### Coefficient of Determination (R^2^)

An alternative way to measure the deviation between the interpolation prediction/kernel density estimation and the mean observed value per grid value is to calculate the coefficient of determination (R^2^) [Eq. 3].

3 $${R}^{2}=1-\frac{\sum {({y}_{i}-{\hat{y}}_{i})}^{2}}{\sum {({y}_{i}-{\bar{y}}_{i})}^{2}}$$

Where: ${y}_{i}$ is the exact gridding value extracted from the original data, ${\bar{y}}_{i}$ is the mean of the exact gridding value, and ${\hat{y}}_{i}$ is the interpolated value. Lower R^2^ indicates higher deviation.

We identified that some of the interpolated layers (n = 8, from 15 layers with no time series, e.g. SDI, species richness, and shear strength—Fig. 5[C]; n=2, from 9 layers with time series based on the mean value for all years, i.e. SDI and species richness—Fig. 5[D]) were found with R^2^ > 0.6. Those with lesser R^2^ include n = 7, from 15 layers of no time series, e.g. BPc, mean body mass, and biomass—Fig. 5[C]; n = 7, from 9 layers with time series based on the mean value for all layers, e.g. Mi, mean body mass, and biomass—Fig. 5[D]. While only a few of the kernel density layers (none from layers with non time series—Fig. 6[C]; n = 3, from 16 layers with time series, i.e. noises from seismic airguns and explosion, and sub-bottom profiler—Fig. 6[D]) are found with R^2^ > 0.6. The others, such as heritage assets and offshore infrastructure from the no time series layers—Fig. 6[C]; and AIS track vessels, other noises, and satellite observation of offshore infrastructures/vessels for layers with time series—Fig. 6[D], were found to have lower R^2^.

We provide the spatial distribution of the RSE and RMSE on each grid for each interpolated and density estimation on each generated layer in the open GIS dashboard https://storymaps.arcgis.com/collections/0f2956eee9704625b74d5cc6157a879d. R^2^ for each grid cannot be calculated.

**Fig. 7:**
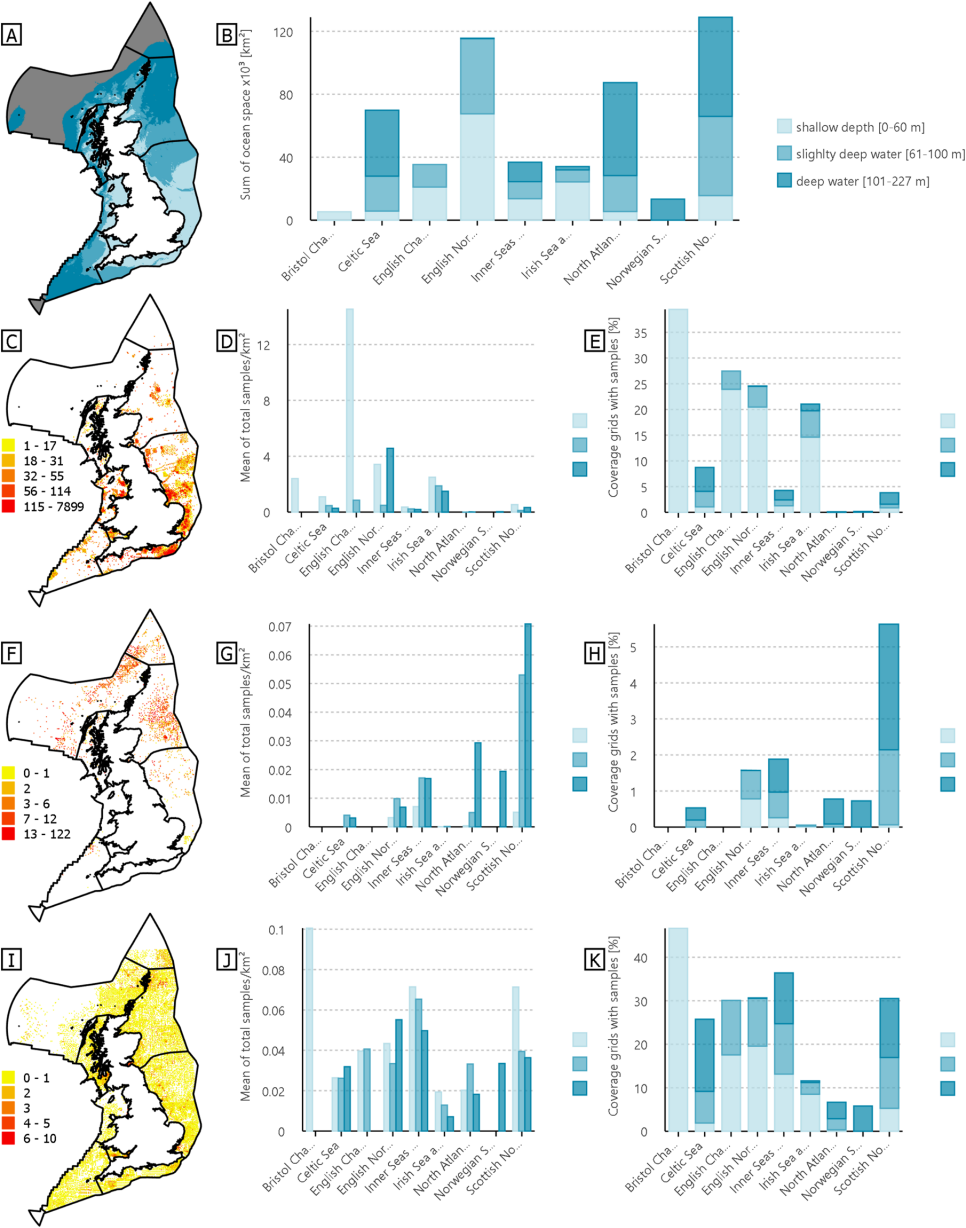
Distribution of input data for interpolated layers (data Type I) in the UK waters. [**A**] classification of water depth in the UK waters – depth up to 227 m are highlighted as they represent the 90^th^ percentile of areas with high human activities^60^. [**B**] Total ocean space per depth classification across different sea regions. [**C,F,I**] Maps showing distribution of benthic [D-E1], geotechnical [D-G1], and seabed [D-G2] sampling data, respectively, with colour bar exhibiting different amounts of samples with natural breaks interval. [**D,G,J**] Charts showing the mean total number of benthic, geotechnical, and seabed samples per km^2^ per sea region, by depth classification. [**E,H,K**] Charts showing the mean percentage of 10 km^2^ grids covered by benthic, geotechnical, and seabed data per sea region, by depth classification.

As the reliability of the interpolated layers is dependent on the number of samples within the area, we provide the spatial mapping of the number of samples, or amounts, found across the UK-EEZ for each input data in Fig. 7. This summary includes the number of sampling data distributions from benthic ecological, geotechnical, and seabed themes across the UK-EEZ that have undergone a spatial interpolation processing method.

## Usage Notes

This study focuses primarily on spatial integration, although some of the collated datasets span time periods, and these differ across datasets, reflecting the most complete and accessible data available for each activity—see Supplementary Information 3 for the time discrepancies between layers with time series. These temporal differences should be considered when interpreting spatial overlaps or cumulative patterns, particularly where ecological responses may be time-sensitive or exhibit lags^19^. Future analytical approaches could benefit from harmonising time windows—for example, by trimming datasets to a common time frame (e.g., 2016–2019), standardising data across years (e.g., through multi-year averaging or normalisation), or including time as a covariate in statistical models—to better account for potential temporal variation and reduce the risk of autocorrelation.

## Supplementary information

Supplementary Information 1

Supplementary Information 2

Supplementary Information 3

## Data Availability

All the spatial data processing methods (spatial interpolation, kernel density estimation, and data extraction to resampling) are routines embedded within commercially available software (ArcGIS, version 3.3). No specific custom code has been used for the spatial data integration process. A catalogue of data sources used is open access in Supplementary Information 2.
