# Supplementary material for: Integrated geospatial datasets to inform marine spatial planning and impact assessment in waters surrounding the United Kingdom
Source: Sci Data. 2025 Nov 20;12:1845. doi: 10.1038/s41597-025-05950-5 (PMC12635140; doi:10.1038/s41597-025-05950-5)
Supplement: Supplementary file 1 — Supplementary Information 1 [file 41597_2025_5950_MOESM1_ESM.pdf]

Welcome to Supplementary Information 1—Detail Methodology for paper:

Title:

Integrated geospatial datasets to inform marine spatial planning and impact assessment in waters surrounding the United Kingdom

Authors:

Hugo Putuhena[a], Thomas J Williams[b], Fraser Sturt[c], David White[a], Martin Solan[b], Jasmin A Godbold[b], Susan Gourvenec[a]

Affiliations:

[a]Civil, Maritime, and Environmental Engineering, Boldrewood Innovation Campus, University of Southampton, Burgess Road, Southampton SO16 7QF, UK
[b]Ocean and Earth Science, National Oceanography Centre Southampton, University of Southampton, Waterfront Campus, European Way, Southampton SO14 3ZH, UK
[c]Archaeology, Avenue Campus, University of Southampton, Highfield Road, Southampton SO17 1BF, UK

ORCID ID:

HP, 0000-0003-1947-6984; TJW, 0000-0002-6616-955X; FS, 0000-0002-3010-990X; DW,0000-0002-2968-582X; MS, 0000-0001-9924-5574; JAG, 0000-0001-5558-8188; SG, 0000-0002-2628-7914

Corresponding author: Hugo Putuhena <H.S.Putuhena@soton.ac.uk>

Introduction

In order to identify candidate correlates relevant to anthropogenic-environment interactions, heritage and other related marine assets, features and activities, we extracted, generated, or resampled spatially resolved layers from 35 sources (listed in Table 1 and *Sheet_1* in Supplementary Information 2) and integrated those layers into a single repository (https://storymaps.arcgis.com/collections/0f2956eee9704625b74d5cc6157a879d). Specifically, the original spatial datasets (listed in Table 1) were drawn from –anthropogenic (12 datasets), ecological (4 datasets), geoscience (11 datasets), and met-ocean (8 datasets) themes. From these sources, we were able to compile or derive a total of 337 layers (100 anthropogenic layers, 123 ecological layers, 94 geoscience layers, and 20 met-ocean layers, *Sheet_3* in Supplementary Information 2). These layers were subsequently harmonised and joined to populate each ~10km^2 (i.e. side length 3.16km) – an adequate griding size for regional analysis. The gridding of each ~10km^2 was made in ED50 UTM 30N projection – which has accuracy 1-5m, sufficient for regional scale mapping (https://epsg.io/23030).

1. Data collection and categorisations

Data sources used to generate the layers for the integrated geospatial dataset included different data formats and standards (Sheet_1 in Supplementary Information 2). In broad terms, each data input reflects either (i) the spatial distribution of ocean activities, heritage assets, or seafloor attributes, so as to quantify the cumulative impact of human activities in the surrounding environment, or (ii) the seabed environment or met-ocean conditions that have potential to influence anthropogenic-environment interactions, or constraints on the location or type of offshore development capacity, including downstream activity.

For organisational purposes, and ease of retrieval, each input dataset and the generated layers are classified into four different thematic categories. Input data utilised for ocean activities and heritage assets fall under the anthropogenic theme (All ***[D-A{number}]*** input data and ***[L-A{number}]*** output layers). Those beneficial for identifying ecological parameters, the seabed environment, and met-ocean parameters are classified respectively under the ecological theme (All ***[D-E{number}]*** data and ***[L-E{number}]*** output layers), geoscience theme (All ***[D-G{number}]*** data and ***[L-G{number}]*** output layers) and met-ocean theme (All ***[D-MO{number}]*** data and ***[L-MO{number}]*** output layers). Supplementary Information 2 (Sheet_1 and Sheet_2) provides the full thematic categorisation for each dataset and associated interpolated layers.

To distinguish the processing steps required to generate each derived layer, each input dataset was categorised by the format and type of the original source. Four data format types were defined. Data format type (i) includes point/polyline/polygon vectors, each representing a single, discrete, and specific geographic location with single or multiple unique sets of continuous numeric attribute values. Data format type (ii) includes data in multi-points/polylines/polygons vectors, each representing a single, discrete, and specific geographic location *of a certain single entity*. Data format type (iii) includes data in polygon vectors that each represent a single, discrete, and specific geographic *zonation* with some unique sets of classified attribute values. Data format type (iv) includes data in *raster or gridded polygon vectors* populated with a single or multiple continuous numeric attribute values. See *Sheet_2* in Supplementary Information 2 for categorisation details for each dataset.

Thirteen of the different input data and 75 of the 285 generated layers are attributed to a time dimension with the given unit in years. These layers are either in the anthropogenic theme (e.g. vessel operations, noise, fishing and offshore infrastructure), or the ecological theme (e.g. benthic biodiversity and function parameters, and marine protected areas). Those layers

generated without a time dimension either portray a spatial condition from a single observation in time or a statistical condition across a specific time range (*Sheet_5* in Supplementary Information 2). These layers are either in the anthropogenic theme (e.g., shipwrecks, seabed obstruction, and oil and gas infrastructure), ecological theme (e.g., bioturbation intensity, benthic mixed depth layer), geoscience theme (e.g., geotechnical and seabed data), or met-ocean theme (e.g., water depth, wave, wind, and currents).

2. Data processing and joining

The primary objective of data processing and joining is to extract or generate all relevant layers from the collected input data, combine them, and populate the underlying square grids (@10km^2 resolution) across the UK-EEZ. Each data format type requires the application of different data processing methods to produce the relevant layers. There are eleven different types of layers that can be produced from each data type(Figure 2 in main manuscript), as follows: (i) extracted amount of point/polyline *[R1]*, (ii) extracted attribute value *[R2]*, (iii) kernel density estimation *[R3]* and (iv) squared error *[R3SqE]*, (v) interpolation without limit zone *[R4]* with (vi) the standard error *[R4StE]*, and (vii) squared error *[R4SqE]*, (viii) interpolation with limit zone *[R5]* with (ix) the standard error *[R5StE]*, and (x) squared error *[R5qE]*, and (xi) the resampled value *[R6]*. The key points of the data processing and joining sequence, summarised in Figure 1 in main manuscript, are:

- Data in *data format type (i),* all of which are sampling data (i.e., benthic [D-E1], geotechnical [D-G1], and seabed sampling data [D-G2]), have been extracted and the mean calculated from all sampling points located inside each 10km^2 grid. However, the extracted mean values contain spatial gaps, as the sample separation distance is not uniform and the abundance of samples can be zero in some of the intended 10km^2 grids. To extend the spatial coverage of the available data, we applied a spatial

interpolation process using the empirical Bayesian kriging (EBK) method to each sampling dataset (i.e., layers [R4]). To avoid the interpolation extending too far beyond the known data, we limited the extent of the interpolation zone (i.e., layers [R5]) using kernel density estimation of sampling points >0. The detailed method for producing the EBK spatial interpolation, including the limitation zone, is provided in section 3.

- For each spatially-interpolated layer, we derived a standard error (SE) of each interpolation from the kriging variance, and used this to determine the relative standard error (RSE) layers [i.e., layers R4SE & R5SE]. Specifically, we divided the SE with the interpolation result on each grid to show the uncertainty of the interpolation result. Calculation of the SE and RSE is provided in Technical Validation in the main manuscript.
- Data in data format type (ii) are either observed entities (e.g., shipwrecks [D-A4]) or reported entities (e.g., noises [D-A1], oil and gas infrastructure [D-A6], and vessels [D-A1]). It is important to note that there may be unobservable or unreported data for each entity, and each may not be in a normal, smooth or continuous spatial distribution. Hence, we used kernel density estimation (KDE) to estimate the probability density and create a normalised spatial distribution based on the available data. These layers (i.e., layers [R3]) can be used, for example, to limit the spatial interpolation layers used to generate layers [R5]. See section 4 for further detail on how the density estimation layer is generated.
- We generated additional uncertainty information for each spatial interpolated or density-estimated layer using the relative standard error (RSE) and root mean square error (RMSE) to assess the accuracy of the generated layers. See section Technical Validation in the main manuscript for the procedure used to obtain both.

- For data with format types (iii) and (iv) it was sufficient to directly extract the data into the intended grids. However, data in type (iv) required resampling (Section 5) to harmonise differences in resolution.
- For data with a time dimension attribute (i.e., benthic samples data [D-E1], reported vessels [D-A1] and noises[D-A2], or fishing effort and time[D-A10, D-A12]), the EBK interpolation, CV calculation, KDE, extraction, or resampling was done for each year and extracted to the dataset (e.g., L-E17 to LE-24, L-A01, L-A05 to L-A11, and L-A61 to L-A62, see Table 1). Then, for each attribute value, each year layer was combined to create multidimensional cube data (coordinates and time). From this cube, the mean and slope of the linear trend with time were extracted to generate time dimensional summary layers (i.e., L-A12 to L-A25, Table 1).

All the generated layers were processed from the input data and integrated to populate the intended grids using ArcGIS Pro software (version 3.3).

3. Spatial data interpolation for data in format type (i)

To generate interpolation layers for data format type (i), we used the empirical Bayesian kriging (EBK) method[1]. This method was picked as this interpolation method has been considered more robust compared to others (e.g., ordinary kriging, spline, inverse diverse weight, or natural neighbours) due to the consideration of spatial autocorrelation, local semi-variograms model generation, and allowed uncertainty generation[1–3]. We also conducted a cross-validation to analyse which of the interpolation methods result in the best accuracy in interpolation of each benthic parameter, based on the root mean square error (RMSE) given. This analysis was conducted using Exploratory Interpolation tool in ArcGIS Pro, which the result can be seen in *Sheet_13* in Supplementary Information 2.

During each EBK interpolation run, we applied a K-Bessel detrended semi-variogram model. We opted for K-Bessel because it offers the most flexible and accurate outcome compared to the other semi-variogram models (e.g., Power, Linear, Thin Plate Spline, Exponential, or Whittle) based on it giving the lowest standard error[4–6]. The detrended version was selected to remove the first order trend and to acknowledge systematic and complex changes in values upon the spatial locations of the interpolated data[7]. To enhance prediction for non-Gaussian data distributions, the data was transformed into a Gaussian distribution using either log empirical (for layers with values >0) or empirical (for layers that may contain value <= 0) transformation[1]. The layers that have been gone through data transformation are those that are not normal in distribution. The normality of the data was assessed using D'Agostino-Pearson omnibus test[8,9]. The list of which layers are normal and not, and which layers have negative and zero value or positive only and whether data transformation has been done or not can be seen in *Sheet_11* in Supplementary Information 2.

Other parameters applied in conducting the EBK interpolations include: cell size=10km^2, maximum points per subset=1000, overlay factors=5, semi-variogram model iterations=1000, and neighbour points range=[20-50].

The algorithms and mathematical equations used to generate the prediction and standard error of the prediction are summarised elsewhere[1,4,10].

4. Spatial density estimation for data in format type (ii)

To generate each spatial density estimation layer, we used the kernel density estimation method (KDE)[11]. In each KDE run, the search radius/bandwidth was determined based on Silverman's Rule-of-thumb bandwidth estimation, which is fast and accurate for normal data distribution, but may over smooth non-normal distributed data[12,13.] Nevertheless, we

considered this method sufficient for the current purpose because the observed level of fit achieved for all layers as represented by the root mean square error (RMSE) between the density estimation and the observed value for all layers. For input data with time-dimensional attributes, we used a single bandwidth reference from the latest year for each KDE run for every year. For example, to generate KDE layers of (i) vessels per year [L-A01], the bandwidth search radius was generated from the year 2019. This allows KDE layers to have a smooth transition through time.

Each KDE output layer was generated for a $10km^2$ grid cell size, and the UK-EEZ boundary was applied as a barrier in the density calculation. Further details on how the application of a barrier affects the KDE calculation are provided by ESRI[13] using the Silverman formula[14].

When generating the KDE layers, with the exception of the noise data, each point represents the appearance of an entity (e.g., a single turbine in the offshore wind turbines dataset D-A03). For noise, the input data is in grids or areal features, as the exact location of the source is unknown. In addition, each grid cell reflects the reported pulse block day, defined as the number of whole days when a certain attribute of noise has been reported. Hence, during each KDE run, we extracted values from the input grids for the number of pulse block days to fill the noise value on each 10 km^2 grid that intersect with the input grids. The intersected $10km^2$ grids values filled with the noise data were then used as the input for the KDE process, and the attributed pulse block days were then used as the population field – or the weight given - for each grid cell.

5. Spatial resampling for data in format type (iv)

Although data type (iv) was available in a readily suitable format (i.e. raster or gridded areal features), we undertook a resampling process to harmonise any differences in resolution between layers. For input data with a resolution lower than $10km^2$, during resampling, we used

the value from the input data co-located with the centre of the intended grid and extracted using the Extract Multi Values to Points tool in ArcGIS pro. While for input data with a resolution higher than $10km^2$, during resampling, we used the spatial join to get the mean from the input data located within the located grid.

References

1. Krivoruchko, K. Empirical Bayesian Kriging. *ArcUser Fall* **6**, 1145 (2012).
2. Hofstra, N., Haylock, M., New, M., Jones, P. & Frei, C. Comparison of six methods for the interpolation of daily, European climate data. *Journal of Geophysical Research Atmospheres* **113**, (2008).
3. Li, J. & Heap, A. D. Spatial interpolation methods applied in the environmental sciences: A review. *Environmental Modelling and Software* vol. 53 173–189 Preprint at https://doi.org/10.1016/j.envsoft.2013.12.008 (2014).
4. Gribov, A. & Krivoruchko, K. Empirical Bayesian kriging implementation and usage. *Science of the Total Environment* **722**, (2020).
5. Kerfoot, W. C. *et al.* Coastal remote sensing: Merging physical, chemical, and biological data as tailings drift onto buffalo reef, lake superior. *Remote Sens (Basel)* **13**, (2021).
6. Katipoğlu, O. M. Analysis of spatial variation of temperature trends in the semiarid Euphrates basin using statistical approaches. *Acta Geophysica* **70**, 1899–1921 (2022).
7. ESRI. What is empirical Bayesian kriging? https://pro.arcgis.com/en/pro-app/latest/help/analysis/geostatistical-analyst/what-is-empirical-bayesian-kriging-.htm (2024).
8. D'agostino, R. B., Belanger, A. & D'agostino, R. B. Jr. A Suggestion for Using Powerful and Informative Tests of Normality. *Am Stat* **44**, 316–321 (1990).
9. Yap, B. W. & Sim, C. H. Comparisons of various types of normality tests. *J Stat Comput Simul* **81**, 2141–2155 (2011).
10. Krivoruchko, K. & Gribov, A. Evaluation of empirical Bayesian kriging. *Spat Stat* **32**, (2019).
11. Węglarczyk, S. Kernel density estimation and its application. *ITM Web of Conferences* **23**, 00037 (2018).
12. Heidenreich, N. B., Schindler, A. & Sperlich, S. Bandwidth selection for kernel density estimation: A review of fully automatic selectors. *AStA Advances in Statistical Analysis* vol. 97 403–433 Preprint at https://doi.org/10.1007/s10182-013-0216-y (2013).
13. ESRI. How Kernel Density works. https://pro.arcgis.com/en/pro-app/latest/tool-reference/spatial-analyst/how-kernel-density-works.htm (2024).

14. Silverman, B. W. *Density Estimation for Statistics and Data Analysis*. (Routledge, 1986).
