# Supplementary material for: Integrated geospatial datasets to inform marine spatial planning and impact assessment in waters surrounding the United Kingdom
Source: Sci Data. 2025 Nov 20;12:1845. doi: 10.1038/s41597-025-05950-5 (PMC12635140; doi:10.1038/s41597-025-05950-5)

Welcome to Supplementary Information 3—Additional Figures for paper:

Title:

Integrated geospatial datasets to inform marine spatial planning and impact assessment in waters surrounding the United Kingdom

Authors:

Hugo Putuhena[a], Thomas J Williams[b], Fraser Sturt[c], David White[a], Martin Solan[b], Jasmin A Godbold[b], Susan Gourvenec[a]

Affiliations:

[a]Civil, Maritime, and Environmental Engineering, Boldrewood Innovation Campus, University of Southampton, Burgess Road, Southampton SO16 7QF, UK
[b]Ocean and Earth Science, National Oceanography Centre Southampton, University of Southampton, Waterfront Campus, European Way, Southampton SO14 3ZH, UK
[c]Archaeology, Avenue Campus, University of Southampton, Highfield Road, Southampton SO17 1BF, UK

ORCID ID:

HP, 0000-0003-1947-6984; TJW, 0000-0002-6616-955X; FS, 0000-0002-3010-990X; DW,0000-0002-2968-582X; MS, 0000-0001-9924-5574; JAG, 0000-0001-5558-8188; SG, 0000-0002-2628-7914

Corresponding author: Hugo Putuhena <H.S.Putuhena@soton.ac.uk>

Supplementary Figure.1. A selection of twelve generated layers from anthropogenic (ocean activities) theme of the integrated dataset. Notations on what layer is represented, as well as the unit and colour bar interval type for each map, are given in the figure. The data source and further details of each map are provided in **Supplementary Information 2**. (Abbreviations: km – kilometers, GW – gigawatt, and kwh – kilowatt-hour).

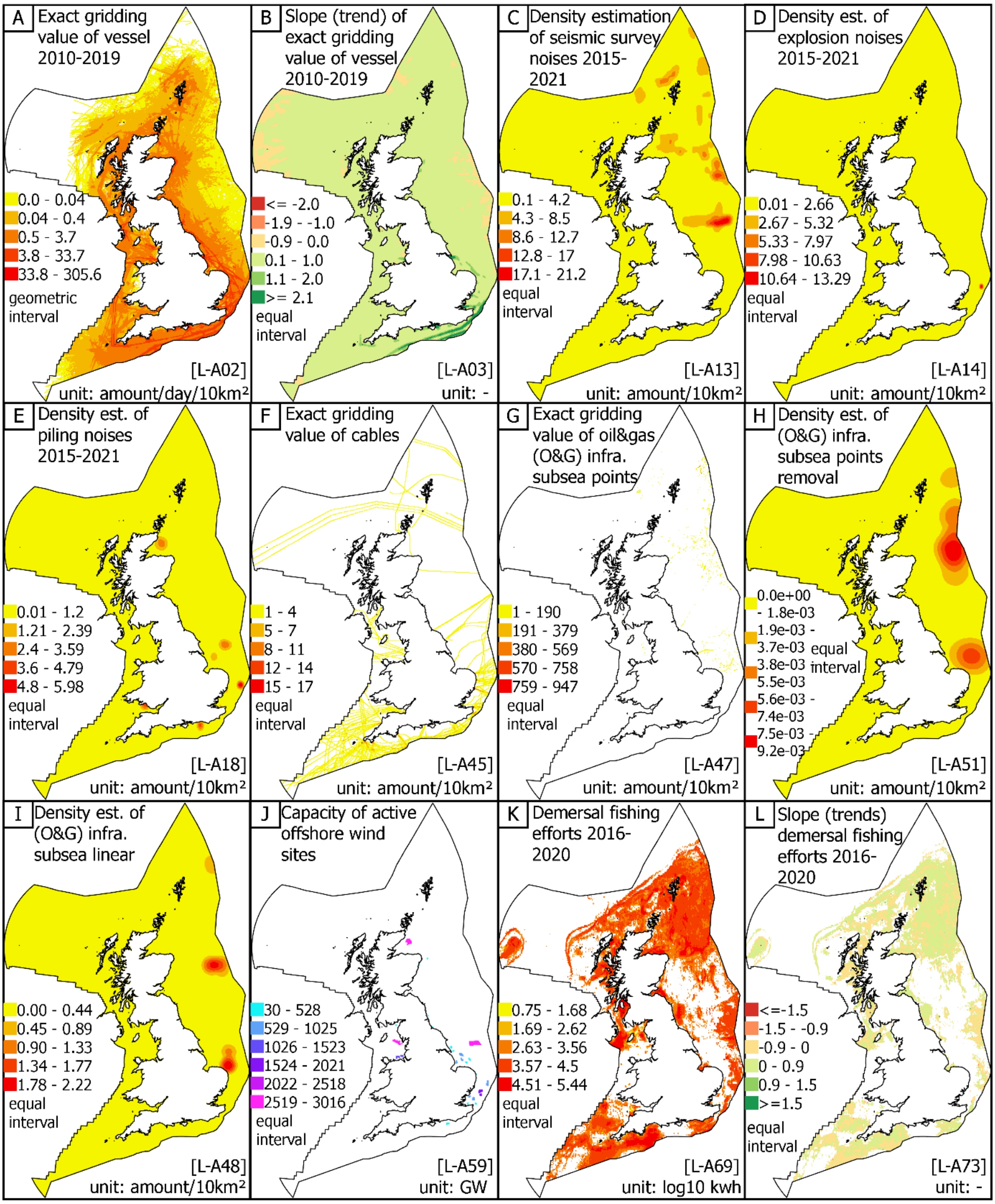

Supplementary Figure.2. A selection of five generated layers from anthropogenic (heritage assets) theme of the integrated dataset. The data source and further detail of each map are provided in Supplementary Information 2. (Abbreviation: kya—kilo years ago).

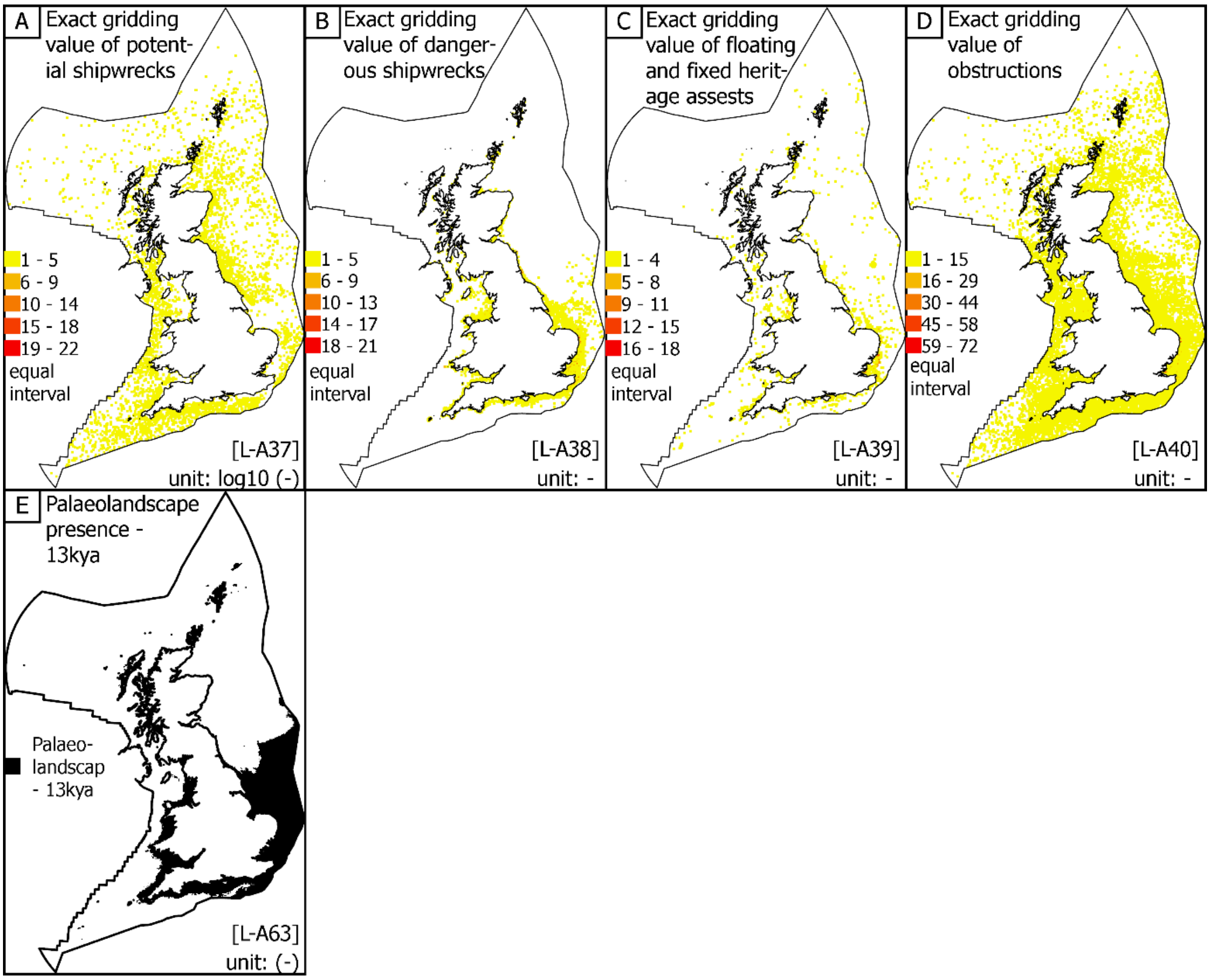

Supplementary Figure.3. A selection of twelve generated layers from ecological theme of the integrated dataset. The data source and further details of each map are provided in **Supplementary Information 2**. (Abbreviation: BPc—Bioturbation index, SDI–Shannon Diversity Index, Mi–mean mobility mode, Ri–mean reworking mode, SE–species evenness, SR–species richness, TA–total abundance).

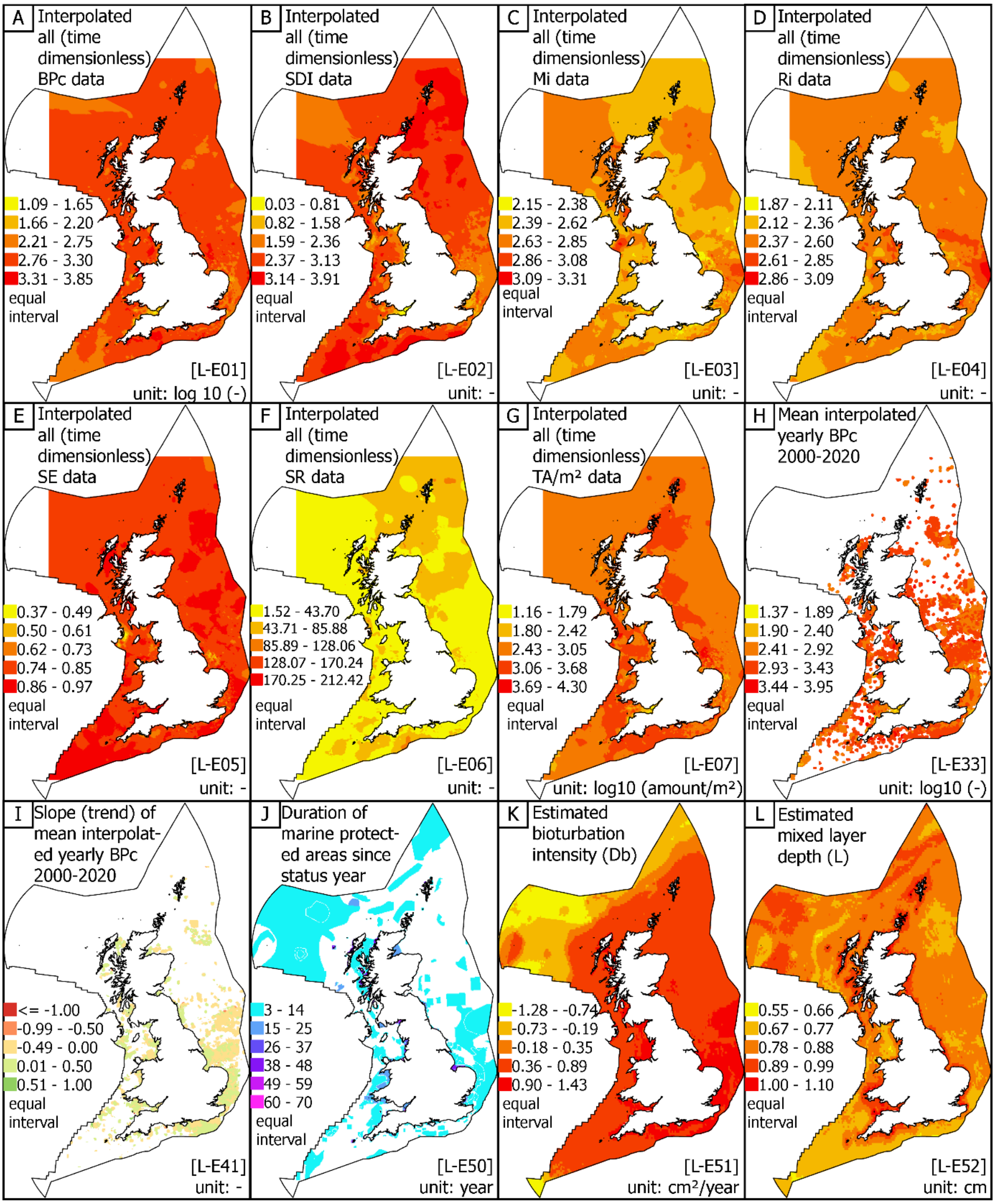

Supplementary Figure.4. A selection of twelve generated layers from geoscience (seabed environment) theme of the integrated dataset. The data source and further detail of each map is provided in Supplementary Information 2. To note, grey colours in [A] and [B] indicate negative values from interpolation due to the presence of original data that also contain negative values, whereby negative values are physically impossible for both [A] and [B] parameters.

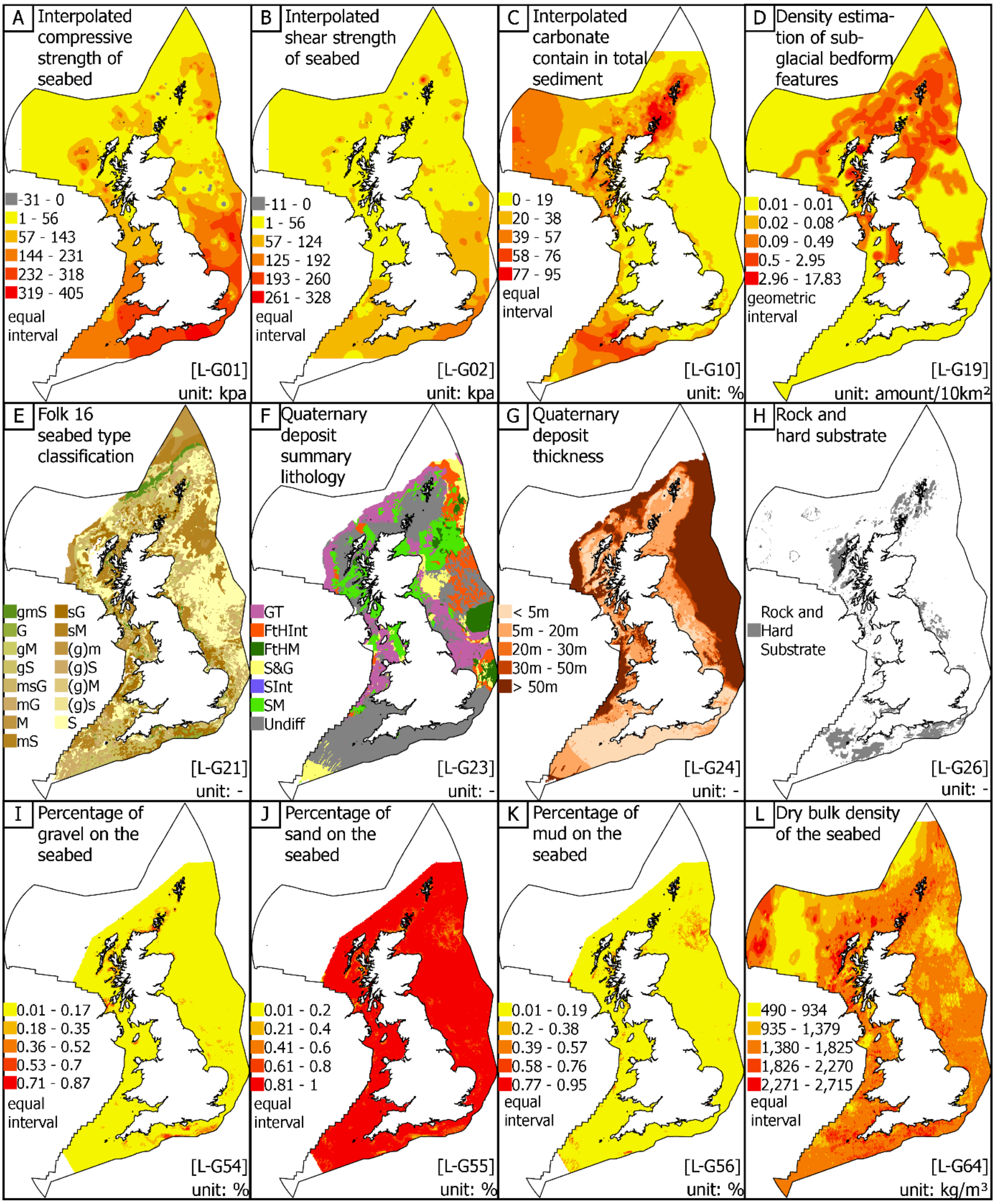

Supplementary Figure.5. A selection of twelve generated layers from met-ocean theme of the integrated dataset. The data source and further detail of each map is provided in **Supplementary Information 2**.

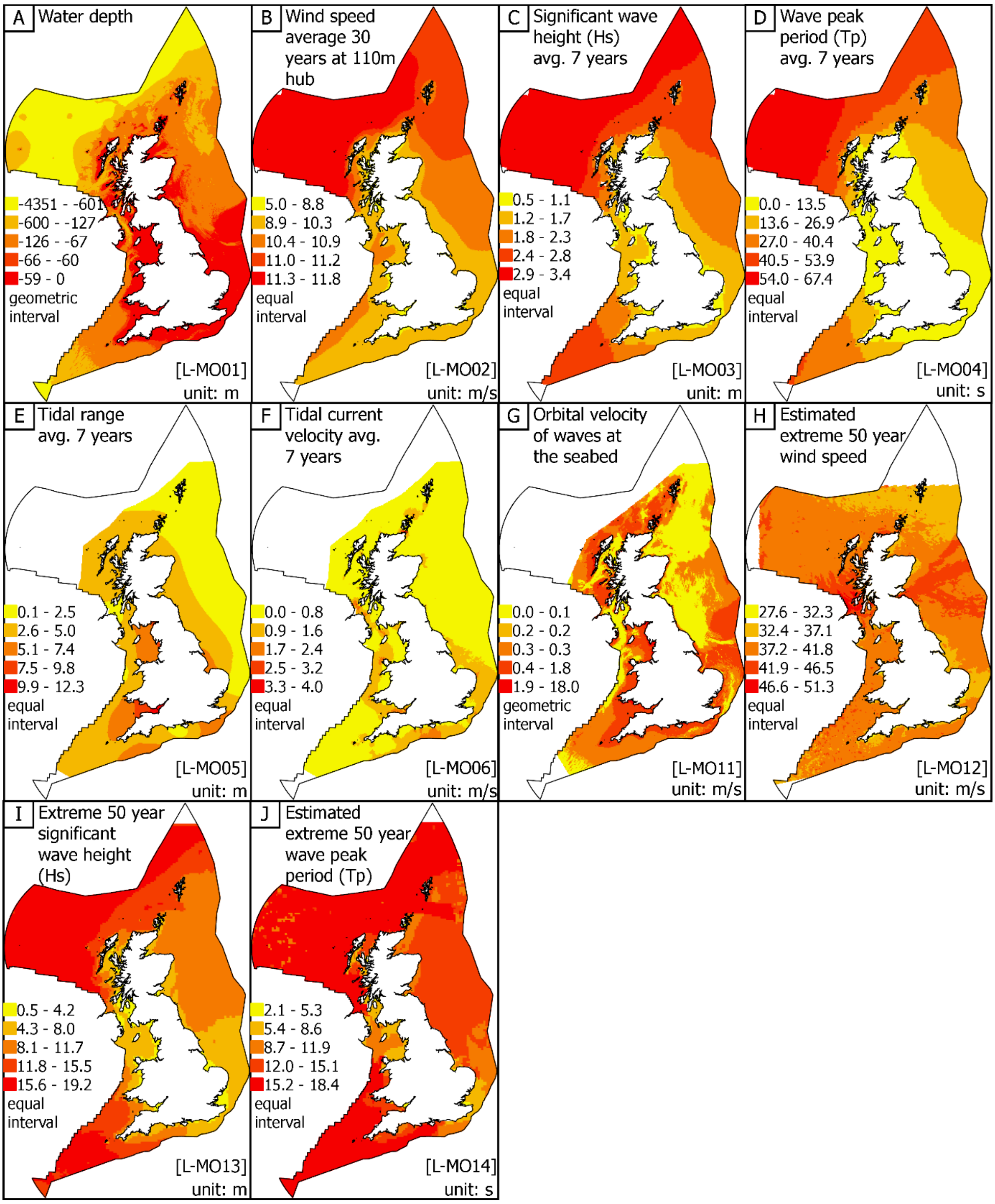

Supplementary Figure.6. Available time windows for each layer with time series.

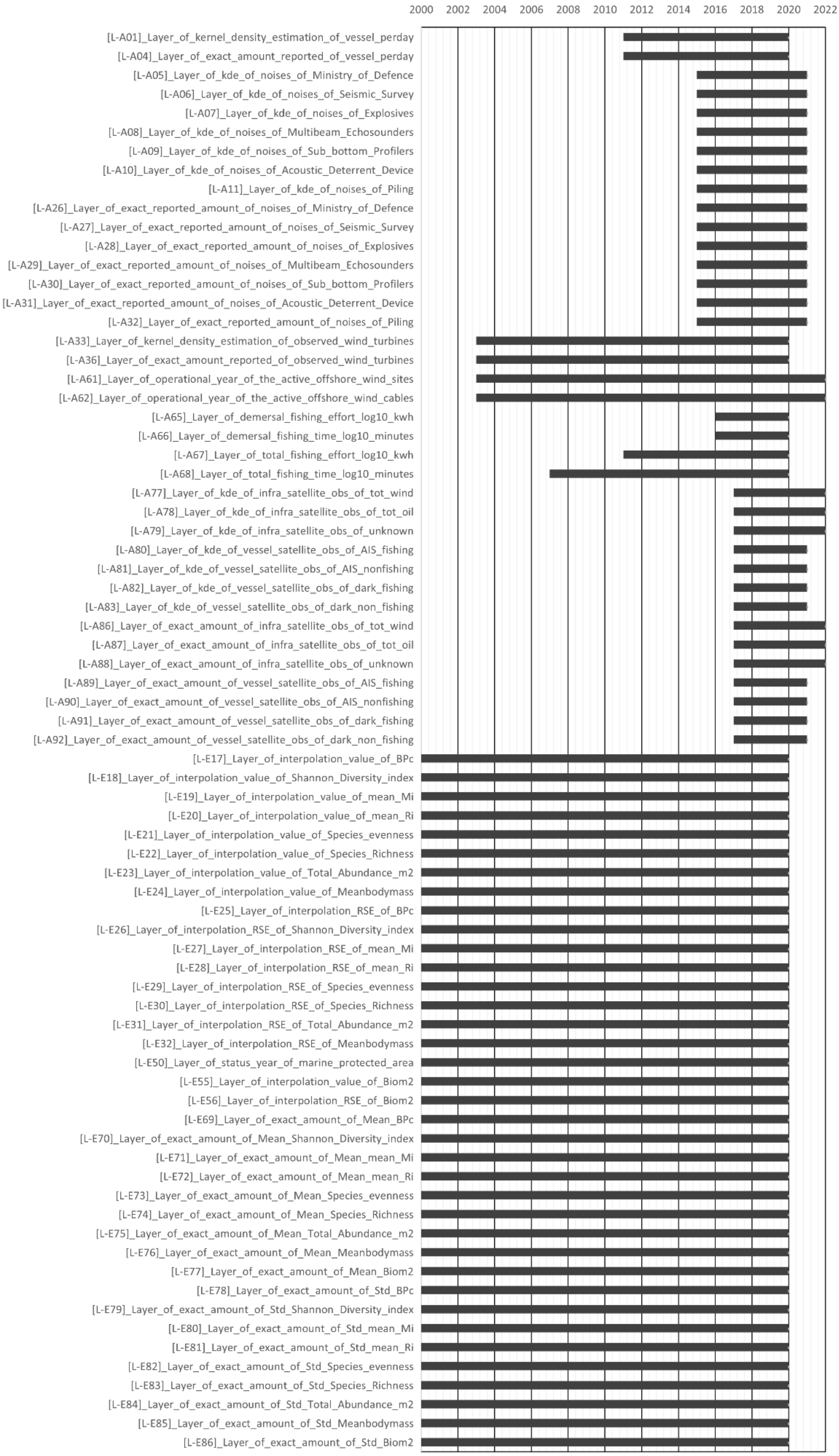

Supplement: Supplementary file 3 — Supplementary Information 3 [file 41597_2025_5950_MOESM3_ESM.pdf]
